# Ovariotomy Following Incision and Long-Drainage

**Published:** 1880-04

**Authors:** 


### OVARIOTOMY FOLLOWING INCISION AND LONG-DRAINAGE.

Mr. Knowsley Thornton read a paper before the Medical Society of London on the above subject, giving a record of three cases of ovarian tumor, in which exploratory incision followed by drainage of the cyst with injections had been employed for two of the cases, and injections for the third. In all re-growth took place, and the author removed all successfully by ovariotomy. The periods between the first operation and the successful ovariotomies were respectively four years, three and one-half years, and eleven years. All these operations were complicated by serious adhesions. The cases taken together teach two important lessons: (1) the thoroughly unsatisfactory nature of the so-called cure by tapping or drainage; (2) the important fact that cases which have been treated by this unscientific method may still be successfully dealt with by the major operation of ovariotomy, even though many years have elapsed since the supposed cure.—*London Lancet, March, 1880.*

